# Disinfection of Ready-to-Eat Lettuce Using Polyhexamethylene Guanidine Hydrochloride

**DOI:** 10.3390/microorganisms8020272

**Published:** 2020-02-17

**Authors:** Jiayi Wang, Yougui Yu, Yuemei Dong

**Affiliations:** 1 College of Food and Chemical Engineering, Shaoyang University, Shaoyang 422000, China; jiayiwangsyau@syau.edu.cn; 2 Shijiashike Co., Ltd., Liaoyang 111000, China; dym662222@163.com

**Keywords:** polyhexamethylene guanidine hydrochloride, household sanitizer, disinfection

## Abstract

As a novel and safe sanitizer, polyhexamethylene guanidine hydrochloride (PHMG) has been used to inhibit the spoilage of agricultural products caused by fungi. However, little is known about its antibacterial effects on vegetables. In this study, we evaluated the disinfection efficacy of PHMG on ready-to-eat lettuce. PHMG (150–200 mg/L) treatment for 5 min was optimal for lettuce disinfection. Compared to several household sanitizers (vinegar: 1% acetic acid; kettle descaler: 1% citric acid; “84” disinfectant: 200 mg/L sodium hypochlorite), PHMG showed the greatest reductions in *Escherichia coli* O157:H7, *Listeria monocytogenes*, aerobic mesophilic counts, aerobic psychrotrophic counts and molds and yeasts. Quality analysis of color (as determined by L*, a* and b*) and determination of electrolyte leakage indicated that PHMG did not cause any additional quality loss as compared to other household sanitizers. These results provide a reference for the application of PHMG as a vegetable sanitizer at the ready-to-eat stage.

## 1. Introduction

Leafy vegetables are an essential source of vitamins, minerals and cellulose. In general, thermal processing detrimentally affects the phenolic content and antioxidant activity of commonly consumed vegetables [1]; hence, most vegetables have higher nutritional value when consumed raw. However, fruits and vegetables prepared without thermal processing have a higher risk of pathogenic infection. In 2011 alone, foodborne pathogens caused 9.4 million infections in the United States, accounting for 55,961 hospitalizations and 1351 deaths [2]. In the United States and Europe, 39.5% and 42.6% of foodborne illnesses, respectively, were caused by consumption of fresh produce [3]. Foodborne pathogen contamination is even more common in developing countries. In Brazil, 53.1% and 3.7% of ready-to-eat vegetables in a market were contaminated with *Escherichia coli* and *Listeria monocytogenes*, respectively [4]. In Rwanda, 15% of agricultural products were found to be contaminated with pathogenic bacteria, of which *E. coli* accounts for the largest proportion (6.1%) [5].

Ready-to-eat vegetables are mainly disinfected by minimal processing at an industrial scale or by consumers in homes or restaurants. Novel disinfection technologies have recently been developed for fresh produce, such as cold plasma, pulsed light and biophage [6]. Despite the good results obtained using these methods, these technologies are limited for use at the ready-to-eat stage, which requires low-cost, easy, nontoxic and consumer-friendly methods [7].

Polyhexamethylene guanidine hydrochloride (PHMG) is a member of the polymeric guanidine family and shows low toxicity. It has broad in vitro bactericidal activity against Gram-positive and Gram-negative bacteria as well as fungi [8]. Its main antibacterial mechanism is collapse of the outer membrane structure, leading to the formation of a pore across the membrane [9,10] and subsequent DNA damage [11]. Accordingly, PHMG has been used to prepare transparent and nonleaching films with antibacterial activity to inhibit pathogenic biofilm formation [12,13]. Regarding disinfection of the produce, PHMG has primarily been used to control fungi. Mathurin et al. [14] found that PHMG has minimal inhibitory concentrations of 0.01–1.9 mg/mL against fungi isolated from cocoa beans. Koffi-Nevry et al. [15] observed that PHMG has strong antifungal activity against species in the genera *Mucor*, *Botrytis*, *Penicillium*, *Geotrichum*, *Aspergillus* and *Colletotrichum* isolated from papaya. Recently, Olemedo et al. [16] found that immersing lemons in 500 mg/L PHMG for 30 s can completely prevent decay caused by *Penicillium digitatum*, with a significantly higher disinfection efficacy than that of the commonly used sodium hypochlorite, even in the presence of 1% organic matter. In 2009, PHMG was approved as a food sanitizer (can directly contact the food material) by the National Health Commission of the People’s Republic Health of China [17]. Methods for producing PHMG have been developed, resulting in a white powder (e.g., Hipoly) or aqueous solution (e.g., Akacid plus), which are safe to use and are easily available in the market. Since the powder has a strong water absorption ability and mildly stimulates the respiratory tract, a tablet form (e.g., Shikean) has been developed that is consumer friendly, easier to store and suitable for household use. However, few studies have evaluated the use of PHMG for the disinfection of bacteria on ready-to-eat vegetables. The objective of this study was to compare the efficacy of PHMG with that of typical household sanitizers (which can be applied to fresh produce) for disinfecting foodborne pathogens and naturally present microorganisms on ready-to-eat vegetables. Green leaf lettuce, commonly eaten raw and characterized by a fragile texture, was selected as the experimental vegetable. 

## 2. Materials and Methods

### 2.1. Lettuce Preparation

Green leaf lettuce (*Lactuca sativa* L. var. *crispa*, without mechanical damage) was purchased from a local market and rinsed with tap water for 30 s to remove dirt. The stem, two outer leaves and inner baby leaves were removed, and the remaining portion was cut into pieces using a circle knife (diameter 5.2 × 10^−2^ m). Each piece was divided into four parts and dewatered using a sterilized manual salad spinner [18].

### 2.2. Lettuce Inoculation

Pathogen inoculation was performed as described in our previous reports [19,20]. Briefly, nontoxic *E. coli* O157:H7 (NCTC12900) and *L. monocytogenes* (ATCC19115) were stored in glycerol solution at a ratio of 1:1. Before each use, *E. coli* O157:H7 and *L. monocytogenes* were purified using sorbitol MacConkey agar (SMAC; Hopebio, Qingdao, China) and *Listeria* chromogenic agar (Land Bridge, Beijing, China), respectively. A single colony was inoculated into tryptic soy broth medium (Hopebio) and shaken overnight to prepare a working solution. The concentration of the bacterial suspension was adjusted to 10^9^ CFU/mL, and 5 mL of the adjusted suspension was mixed with 200 mL of a sterilized 0.85% sodium chloride (NaCl) solution in a sterilized plastic sampling bag. Next, 10 g of lettuce leaves was placed in the bag and manually massaged for 5 min. The inoculated sample was placed in a sterilized plastic tray in a biosafety cabinet for air drying. The sample containers were sealed and placed at 4 °C for 24 h to obtain final *E. coli* O157:H7 and *L. monocytogenes* counts on lettuce of 10^6^–10^7^ CFU/g.

### 2.3. Disinfection

Common household sanitizers, including white vinegar (Haitian, Foshan, China), kettle descaler (Lvsan, Beijing, China) and “84” disinfectant (Blue Moon, Guangzhou, China), were used for comparisons of disinfection efficacy with that of PHMG (Shijiashike, Liaoyang, China). The active compound, concentration and contact time for each are shown in Table 1. Untreated samples were used as a control group. Sanitizer was diluted with tap water (20 ± 2 °C) to prepare the disinfection solution. The free chlorine concentration was adjusted as desired using a Free Chlorine Test Kit (Lohand, Hangzhou, China). Thirty grams of inoculated lettuce was immersed in disinfection solution at a ratio of 1:20 [7]. After disinfection, the samples were transferred to tap water for 30 s to remove the residual sanitizer. 

### 2.4. Microbiological Analysis

After disinfection, the samples (15 g) were transferred to a sterilized stomacher bag, diluted 1:15 in 225 mL of sterilized 0.85% NaCl solution and homogenized in a stomacher for 90 s. For *E. coli* and *L. monocytogenes*, 0.1 mL of diluted bacterial suspension was surface-plated on SMAC agar and *Listeria* chromogenic agar, respectively, and microbial counts were obtained after a 24-h incubation period at 37 °C. For naturally present microbial taxa, 0.1 mL of the diluted bacterial suspension was surface-plated on rose Bengal agar (Hopebio) and incubated at 30 °C for three days to quantify molds and yeasts (M&Y). Additionally, 1 mL of the dilution was pour-plated onto plate count agar (Hopebio) and incubated at 37 °C for 2 days to obtain aerobic mesophilic counts (AMC) and at 7 °C for 10 days to obtain aerobic psychrotrophic counts (APC).

### 2.5. Color Measurement and Electrolyte Leakage Analysis

Instrumental color was analyzed using a colorimeter (CR400; Konica Minolta, Osaka, Japan) with an aperture diameter of 11 mm. The illuminant was D65 and the color space used was a CIELab system. After disinfection, ten pieces of the sample were randomly selected from each group, and two sites per piece were analyzed for a total of 20 readings per replicate. The values of L*, a* and b* were recorded for analysis. For L*, a* and b*, negative to positive values represented dark to light, green to red and blue to yellow, respectively. The colorimeter was calibrated using a white standard plate (*Y* = 82.80, *x* = 0.3194, *y* = 0.3264) before each use [19].

The extent of damage to the lettuce after disinfection was evaluated by measuring electrolyte leakage as described by Wang et al. [20], with minor modifications. Five grams of the sample was immersed in 250 mL distilled water for 20 s to remove the sanitizer residue, which can interfere with conductivity measurements. The samples were placed in 150 mL distilled water, and conductivity was measured after 30 min. After standing for 12 h at −20 °C, the sample was naturally thawed at room temperature, and then conductivity was measured. Electrolyte leakage was calculated using the following formula:

$$\mathrm{Electrolyte}\;\mathrm{leakage}\;\left(\%\right)=\frac{{\mathrm{Conductivity}}_{30\;\min}}{{\mathrm{Conductivity}}_{12\;h}}$$

### 2.6. Statistical Analysis

Each experiment was performed three times. Differences between group means were evaluated by one-way analysis of variance using SPSS v.20 (IBM lnc., Armonk, NY, USA), and differences (*p* < 0.05) in mean values were analyzed by post hoc Duncan’s multiple range tests. 

## 3. Results and Discussion

### 3.1. Effects of Different PHMG Concentrations and Contact Times on Pathogens Inoculated on Ready-to-Eat Lettuce

A minimal processing time in the produce industry is generally 1–3 min [22,23]. At the ready-to-eat stage, the processing time is not strictly uniform and sometimes even exceeds 5 min [24,25]. In this study, when the contact time was 2 min, the effects of PHMG on *E. coli* O157:H7 did not increase significantly as the concentration was increased from 50 to 100 mg/L (Table 2). However, the reduction in *E. coli* O157:H7 increased significantly from 1.40 to 1.72 log CFU/g as the concentration was increased from 100 to 150 mg/L, and the reduction in *E. coli* O157:H7 did not increase significantly as the concentration was increased from 150 to 200 mg/L. As the treatment time increased from 2 to 5 min, the disinfection effect of PHMG at a high concentration (100–200 mg/L) was significantly improved. However, when the processing time was further increased to 8 min, the disinfection efficacy did not improve. Taken together, 150–200 mg/L PHMG for 5 min was optimal for *E. coli* O157:H7 disinfection. 

Similarly, the disinfection efficacy of PHMG (100–200 mg/L) against *L. monocytogenes* increased significantly as the disinfection time was increased from 2 to 5 min (Table 3). The disinfection efficacy did not improve as the disinfection time was further increased to 8 min (except for the 50 mg/L group). The disinfection efficacy improved significantly when the concentration exceeded 75 mg/L; however, the efficacy did not improve as the concentration was increased from 150 to 200 mg/L. Thus, 150–200 mg/L PHMG for 5 min was appropriate for *L. monocytogenes* disinfection. 

Overall, the disinfection efficacy is relatively low in the concentration range of 50–75 mg/L. This may be explained by the cell membrane adhesion of PHMG; a low concentration of PHMG may only be sufficient to damage the cell membrane and generate pores, whereas a high concentration can allow more substances to enter the cell and damage DNA, therefore enhancing the disinfection efficacy.

### 3.2. Effects of PHMG and Household Sanitizers on Pathogens and Naturally Present Microbes on Ready-to-Eat Lettuce

**Figure 1:**
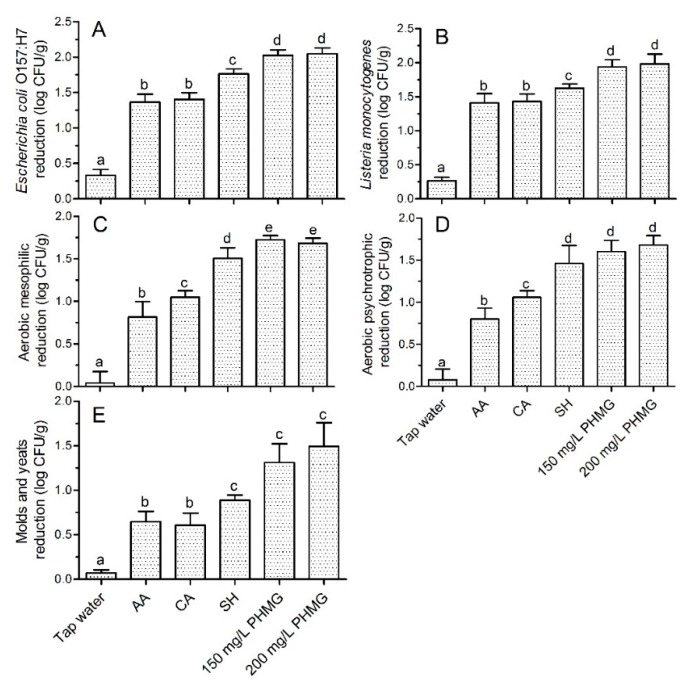
Microbial reduction (log CFU/g) of *E. coli* O157:H7 (**A**), *L. monocytogenes* (**B**), aerobic mesophilic counts (**C**), aerobic psychrotrophic counts (**D**), and molds and yeasts (**E**) present on ready-to-eat lettuce. Bars show means ± standard deviations, and different letters above the columns indicate significant differences (*p* < 0.05). AA: acetic acid; CA: citric acid; SH: sodium hypochlorite.

The *E. coli* O157:H7 count, *L. monocytogenes* count, AMC, APC and M&Y in the control group were 6.70 ± 0.13, 6.75 ± 0.20, 5.97 ± 0.45, 6.02 ± 0.41 and 5.10 ± 0.71 log CFU/g, respectively. Based on analyses of disinfection times and antimicrobial effects, 150–200 mg/L PHMG treatment for 5 min was selected to compare the disinfection efficacy with various household sanitizers (Table 1). Two generally recognized safe sanitizers, citric acid (CA) and acetic acid (AA), are widely used in minimally processed food production and at the ready-to-eat stage [18,22]. In this study, the disinfection efficacies of CA against *E. coli* and *L. monocytogenes* were similar to those of AA (Figure 1A,B). The disinfection efficacies of these two acids were significantly lower than those of sodium hypochlorite (SH). PHMG showed the greatest reductions in *E. coli* and *L. monocytogenes* and was more effective than AA, CA and SH. The differences in microbial reduction caused by these types of sanitizers (organic acids, oxidizing sanitizer and PHMG) may be related to differences in the mechanism underlying the antibacterial effects. The antibacterial mode of action of PHMG was discussed above. The antibacterial effects of organic acids are attributed to the high intracellular pH, which promotes the dissociation of acidic molecules after cell penetration, resulting in anion accumulation in the cell and toxic effects on the cell membrane, acid-sensitive proteins, DNA and RNA [18,20]. Chlorine primarily damages the cell membrane and leads to leakage of cellular macromolecules [26]. Our results indicate that the specific mechanism underlying the antibacterial effects of PHMG is more effective than those of the other sanitizers for disinfecting foodborne pathogens. The use of different sanitizers (with different antibacterial mechanisms of action) is reported to result in additional microbial reductions compared to those obtained using a single type of sanitizer. For example, Wang et al. [20] found that sequential washing of lettuce using lactic acid and aqueous ozone (AO) shortened the processing time by 30 s compared to lactic acid treatment alone, with further reductions of *E. coli* O157:H7 and naturally present microbial taxa. Combined use of CA and AO reduces *E. coli* and *L. monocytogenes* counts on lettuce and mushrooms to a greater extent than either agent alone [27,28]. Recently, slightly acidic electrolyzed water has gained attention because of its strong antimicrobial activity [6,29]. Based on the different antibacterial mechanisms, it may be useful to combine citric acid with PHMG for disinfection; further studies should focus on the development of an optimized powder containing PHMG and CA.

With respect to naturally present microbial taxa, the disinfection efficacies of PHMG against AMC, APC, and M&Y were greater than those of AA and CA (Figure 1C–E), whereas its activity levels against APC and M&Y were not significantly higher than those of chlorine (Figure 1D,E). This may be because of the diversity of bacterial species present on the lettuce; the different membrane structures among bacteria have different levels of resistance to PHMG. Overall, PHMG resulted in the greatest microbial reduction compared to household sanitizers.

### 3.3. Effects of PHMG and Household Sanitizers on Electrolyte Leakage and Color of Ready-to-Eat Lettuce

**Figure 2:**
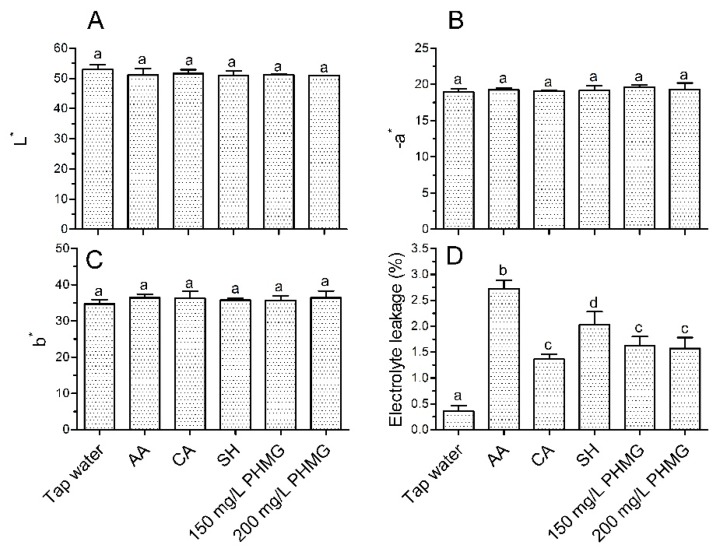
Effects of various treatments on instrumental color (**A**–**C**) and electrolyte leakage (**D**) of ready-to-eat lettuce. Bars show means and standard deviations, and different letters above the columns indicate significant differences (*p* < 0.05). AA: acetic acid; CA: citric acid; SH: sodium hypochlorite.

Based on analyses of the L*, a* and b* values, PHMG and several household sanitizers did not negatively influence the color quality of lettuce compared to values obtained using tap water (Figure 2A–C). We found that 1% AA led to the highest electrolyte leakage, with significantly higher values than in the other groups (Figure 2D). As the AA concentration was increased to 1.9% and the washing time was increased to 10 min, the sensory qualities including overall acceptability, odor and texture were negatively affected [25]. Wang et al. [30] showed that washing with 1% AA for 2 min did not negatively affect the sensory color and texture of ready-to-eat lettuce, indicating that the washing time and concentration of AA were the major factors influencing the quality of lettuce. Moreover, a loss of color quality was observed after storage for several days. For example, in our previous study, a loss of visual quality was observed in lettuce after washing with 1% AA and storage for 5 days [19]. The electrolyte leakage of PHMG was similar to that of CA and significantly lower than that of AA and SH (Figure 2D), indicating that PHMG did not lead to additional surface damage of the lettuce compared to other household sanitizers.

## 4. Conclusions

Our results support the use of the novel sanitizer PHMG for effectively disinfecting fresh produce. The disinfection efficacy of PHMG was higher than that of typical household sanitizers, without negatively affecting instrument color. These results provide a reference for the use of PHMG to disinfect fresh produce at the ready-to-eat stage. Interestingly, the disinfection efficacy of PHMG was highest at an intermediate concentration range of 100–150 mg/L. We will explore the reasons for these changes in future studies using molecular methods (e.g., omics techniques).

## Figures and Tables

**Table 1:** Disinfection treatments for ready-to-eat lettuce.

Household Sanitizer	Active Compound	Concentration Used *	Contact Time (min)	References
Tap water			5	
Vinegar	Acetic acid	1%	5	[21]
Kettle descaler	Citric acid	1%	5	[21]
“84” disinfectant	Sodium hypochlorite	200 mg/L	5	[7]
PHMG		150 mg/L	5	
		200 mg/L	5	

* Concentrations are expressed basing on the active compound.

**Table 2:** Microbial count reduction (log CFU/g) of *Escherichia coli* O157:H7 after washing with different concentrations of polyhexamethylene guanidine hydrochloride (PHMG).

Contact Time (min)	Sanitizer Concentration (mg/L)				
	50	75	100	150	200
2	1.21 ± 0.15 ^Aa^	1.37 ± 1.40 ^Aa^	1.40 ± 0.08 ^Aa^	1.72 ± 0.07 ^Ab^	1.76 ± 0.13 ^Ab^
5	1.45 ± 0.07 ^ABa^	1.52 ± 0.13 ^Aa^	1.64 ± 0.11 ^Ba^	1.97 ± 0.05 ^Bb^	1.99 ± 0.14 ^ABb^
8	1.59 ± 0.24 ^Ba^	1.56 ± 0.37 ^Aa^	1.69 ± 0.14 ^Bab^	2.03 ± 0.13 ^Bab^	2.01 ± 0.15 ^Bab^

Counts in the control group were 6.54 ± 0.31 log CFU/g. Different lowercase letters and different capital letters in the same row and column, respectively, indicate significant differences (*p* < 0.05).

**Table 3:** Microbial count reduction (log CFU/g) of *Listeria monocytogenes* after washing with different concentrations of PHMG.

Contact Time (min)	Sanitizer Concentration (mg/L)				
	50	75	100	150	200
2	1.12 ± 0.05 ^Aa^	1.15 ± 0.19 ^Aa^	1.55 ± 0.12 ^Ab^	1.75 ± 0.11 ^Ab^	1.76 ± 0.10 ^Ab^
5	1.37 ± 0.05 ^Ba^	1.51 ± 0.10 ^Ba^	1.76 ± 0.06 ^Bb^	2.01 ± 0.13 ^Bc^	1.94 ± 0.76 ^Bc^
8	1.57 ± 0.07 ^Ca^	1.54 ± 0.07 ^Ba^	1.85 ± 0.05 ^Bb^	2.02 ± 0.08 ^Bc^	1.95 ± 0.08 ^Bbc^

Counts for the control group were 6.73 ± 0.29 log CFU/g. Different lowercase letters and different capital letters in the same row and column, respectively, indicate significant differences (*p* < 0.05).
